# Thyroid abnormalities in children with Down syndrome at St. Paul's hospital millennium medical college, Ethiopia

**DOI:** 10.1002/edm2.337

**Published:** 2022-04-14

**Authors:** Birtukan Mulu, Bereket Fantahun

**Affiliations:** ^1^ Department of Pediatrics Saint Paul's Hospital Millennium Medical College Addis Ababa Ethiopia

**Keywords:** Down syndrome, thyroid abnormality, Ethiopia

## Abstract

**Background:**

Subclinical hypothyroidism (SCH) is the commonest thyroid abnormality in patients with Down syndrome (DS). The purpose of this study was to determine the prevalence and types of thyroid abnormalities, to assess the age at diagnosis, and to examine the screening practice in children with DS in a resource limited setting.

**Methodology:**

A retrospective study was conducted in children with DS seen at endocrine follow‐up clinic. Data were collected from patients' registration book and medical records.

**Result:**

A total of 115 patients with DS were included in the study out of which 64 (59.8%) were males. Their median age at diagnosis was 9 months (range 4–15 years). Thyroid function test (TFT) was done at least once for 107 (93%) patients. Abnormal thyroid function was observed in 51 (47.7%) patients. The commonest thyroid abnormality was SCH (30/107, 28%) followed by congenital hypothyroidism (11/107, 10.3%), overt hypothyroidism (9/107, 8.4%) and hyperthyroidism (1/107, 0.9%). Most of the patients (86/107, 80.4%) were tested initially in the first 2 years of life. From those who were tested between the age of 2–6 months (*n* = 22 patients), seven (31.8%) patients had thyroid abnormalities.

**Conclusion:**

Thyroid abnormalities were seen in a remarkable proportion of DS patients. The detection of abnormalities in the patients with age range of 2–6 months demands the need for additional TFT in this age category apart from the standard recommendation. Early diagnosis and management of thyroid abnormalities are important to decrease further impairment of cognition function in children with DS.

## Graphical abstract

Thyroid abnormalities were seen in a remarkable proportion of DS patients. The detection of abnormalities in the patients with the age range of 2 to 6 months demands the need for additional TFT in this age category apart from the standard recommendation. Early diagnosis and management of thyroid abnormalities are important to decrease further impairment of cognition function in children with DS.

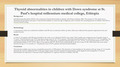

## 1 INTRODUCTION

Down syndrome (DS) is a chromosomal abnormality occurring in children with an estimated incidence of 1/600 to1/800 live births.^1^ Its incidence has increased recently as a result of the increased number of mothers above the age of 35 years who are giving birth.^2^ Diagnosis of DS depends on the finding of a genetic testing karyotype for trisomy.^3^ However, where karyotyping is not routinely done, the diagnosis can be made based on phenotypic features with 88% accuracy.^1^ DS is usually characterized by congenital malformations, medical conditions and cognitive impairments.^4^ The common medical conditions in DS are cardiac abnormalities, thyroid disorders, neurologic abnormalities, hematologic abnormalities and ophthalmic abnormalities.^4^, ^5^, ^6^ Thyroid disorder is one of the abnormalities which can contribute to mental retardation in DS patients.^4^

Depending on the definition of the thyroid abnormalities, the prevalence of thyroid dysfunction in children with DS ranges from 4% to 19.5%.^7^, ^8^, ^9^, ^10^ Its lifetime prevalence in DS is estimated to be 13%–63 %.^11^, ^12^ Thyroid dysfunction in DS can be autoimmune or non‐autoimmune related.^6^ From the autoimmune thyroid disease, Hashimoto's thyroiditis (HT) is more common than Graves' disease. HT might progress to Graves' disease (GD) more frequently in patients with DS than in the general population.^13^, ^14^ There are different forms of thyroid disorders. Congenital hypothyroidism (CH), subclinical hypothyroidism (SCH), overt hypothyroidism and hyperthyroidism.^15^, ^16^ From these, SCH is the most common form of thyroid abnormality reported in 25–32% of DS patients.^17^, ^18^ Non autoimmune SCH showed higher prevalence and earlier onset than autoimmune thyroiditis in children with DS which might be explained by congenital alteration in the regulation of thyroid gland which is directly related to the trisomy of chromosome 21.^19^

Thyroid hormones are vital for the development of the central nervous system, particularly during infancy.^20^ Failure of the thyroid gland to produce an adequate amount of thyroid hormones and delay in diagnosis of hypothyroidism in DS children will result in worsening of psychomotor development, somatic growth and mental retardation.^21^, ^22^ As the symptoms of hypothyroidism overlap with manifestations of DS, routine screening helps to pick thyroid function abnormalities earlier. In this regards, the American Academy of Pediatrics recommends thyroid function screening in children with DS at birth, 6 months, 12 months and yearly.^4^ Therefore, screening for thyroid abnormalities at the recommended interval is crucial.^22^ In many parts of the world, newborn screening for thyroid abnormalities is mandatory.^23^ However, this is not a routine practice in developing countries like Ethiopia. Studies in DS patients were also very scarce in the developing countries.^24^, ^25^ The purpose of this study was to assess the prevalence and types of thyroid abnormalities in children with DS, to determine the age at diagnosis, and to evaluate thyroid function screening practice in children with DS in resource limited setting.

## 2 METHODOLOGY

An institution‐based retrospective cohort study was conducted at St. Paul's Hospital Millennium Medical College (SPHMMC), a tertiary teaching hospital in Addis Ababa, Ethiopia. The SPHMMC has a paediatrics endocrinology clinic. The clinic is run by two consultant paediatric endocrinologists and senior paediatric residents twice per week. Children with DS who had follow‐up at the paediatrics endocrinology clinic from November 2015 to November 2019 were included in the study. Ethical approval was obtained from the institutional review board of SPHMMC. Patient information was anonymized and de‐identified before analysis.

Data on current age, sex, age at first thyroid function tests (TFT) determination, maternal age at conception, TFT (TSH and T4). For the TFT, 2–4 ml whole blood was taken using serum separator tube to analyse TSH and T4 using chemistry machine (Cobas 6000 analyser). Echocardiography and karyotyping results were retrieved from registration book and patient medical charts. The data were entered and analysed using SPSS Version 24. Continuous variables were categorized. Frequency tables and percentiles were used to describe variables. *p*‐value < .05 was considered statistically significant.

## 3 OPERATIONAL DEFINITION

Phenotypic DS was defined as those patients diagnosed by the physical appearance of DS such as, low muscle tone, flat facial features, small nose, upward slanting of the eyes, epicantal fold, small abnormally shaped ears, simian crease, hyper flexibility, evidence of congenital heart disease and hypothyroidism.^1^

Thyroid abnormality classification is indicated in Table 1.

**TABLE 1:** Operational definition

Congenital hypothyroidism	^a^”High” TSH in newborn period and (2–28 days)
Subclinical hypothyroidism	^b^“High” TSH with normal T4 at diagnosis
Overt hypothyroidism	^b^“High” TSH with low T4 at any time
Hyperthyroidism	^b^”LOW” TSH with high T4 level

High TSH (above 10 μIU/ml) or low T4 (below 10 μg/dl) in the new‐born period. In this study new‐born were tested between 7 days and 1 month of age.

Normal reference ranges for TFT beyond the neonatal period in the current study was as follows; for TSH 0.5‐5 μIU/ml and for total T4 5.1–14 mcg/dl. The high and low cut‐off points were based on established reference ranges.^37^, ^38^

Thyroid autoantibody tests and thyroid ultrasound were not done.

## 4 RESULTS

A total of 115 patients with DS were included in the study, out of which 64 (59.8%) were males and 51 (40.2%) were females. Their median age at diagnosis was 9 months (range 4–15 years). DS was confirmed by karyotype examination in 24 (20.9%) patients only and for the remaining 91 patients (79.1%), diagnosis of DS was made by clinical diagnosis (phenotypic DS).

Of the 115 patients, TFT was done at least once for 107 (93%) patients. TFT was not done for eight patients. Thyroid abnormalities were seen in 47.7% (51/107) of the DS patients. Analysis of the types of thyroid abnormalities showed, 28% (30/107) of the DS patients had SCH, 10.3% (11/107) patients were diagnosed with congenital hypothyroidism, 8.4% (9/107) patients had overt hypothyroidism, and only one female patient had hyperthyroidism. The patient with hyperthyroidism was diagnosed at the age of 6 years (Table 2).

**TABLE 2:** Prevalence of different types of thyroid disease in DS patients at SPHMMC, Addis Ababa Ethiopia

Thyroid disease	*N* (%)	Mean Age at diagnosis	Mean TSH at diagnosis μIU/ml (range)	Mean T4 at diagnosis μg/dl (range)
CH	11 (10.3%)	3 weeks (7 days–1 month)	18.8 (^a^8–51)	12.2 (^b^8.4–15)
SCH	30 (28%)	2.48 year	8.58 (5.3–17.9)	10.01 (3.4–18)
TSH 5–10	26 (24.3%)			
TSH >10	4 (3.7%)			
Hyperthyroidism	1 (0.9%)	6 years	0.05	24
Overt HT	9 (8.4%)	2.76 year	10.89 (5–14.3)	7.97 (1.1–13.5)
Normal TFT	56 (52.3%)	1.91 year	3.88 (0.46–5.4)	10.83 (5–23)
Total	107			

TSH was below 8uIU/ml but the ^b^T4 was low 8.4 ug/dl in one patient and it was included as CH and the patient was on levothyroxine.

In our study, out of the 22 DS patients who were screened between the ages of 2–6 months, 31.8% (7/22) had thyroid abnormalities as it was indicated in Table 3.

**TABLE 3:** Age at first TFT determination and Thyroid abnormalities detection in DS patients at SPHMMC, Addis Ababa Ethiopia

Age	TFT done = *n*	Thyroid abnormality detected = *n* (%)	Types of abnormality Detected = *n*
Birth‐1 month	23	13/23 (56.5)	Congenital hypothyrodisim‐11 Subclinical hypothyroidism ‐2
2 month–6 months	22	7/22 (31.8)	Subclinical hypothyroidism‐6 Overt hypothyroidism‐1
6 month‐1 year	20	11/20 (55)	Subclinical hypothyroidism‐7 Overt hypothyroidism ‐4
1–2 years	21	9/21 (42.8)	Subclinical hypothyroidism‐7 Overt hypothyroidism‐2
2–5 years	8	3/8 (37.5)	Subclinical hypothyroidism‐3
5–10 years	10	6/10 (60)	Subclinical hypothyroidism‐4 Overt hypothyroidism‐1 Hyper‐1
10–15 years	3	2/3 (66.6)	Subclinical hypothyroidism 1 Overt hypothyroidism‐1
Total	107	51/107 (47.7)	

Boys were affected with thyroid abnormalities predominantly (33/51, 64.7%) than girls (18/51, 35.3%) but statistical difference was not observed in terms of mean TSH, T4 and types of thyroid abnormalities between the two groups.

We analysed the maternal age at conception, and we found more than half (62/115, 53.9%) of the mothers were older than 35 years of age when they got pregnant with these children (Table 4).

**TABLE 4:** Demographics and comorbid diagnoses DS patients at SPHMMC, Addis Ababa Ethiopia

Frequency and percentage		
	*N*	%
Mean age, years (range)	3.48 (0.08–15)	
Gender		
Male	69	60
Female	46	40
Maternal age		
18 to 35	53	46.1
>35	62	53.9
Karyotype done		
yes	24	20.9
No	91	79.1
Congenital cardiac disease		
No	63	54.8
Yes	52	45.2
Visual impairment	26	26.6
Sleep apnoea	9	7.8
Seizure disorder	7	6.1
Autism spectrum disorder	4	3.5
Hirschsprung disease	1	0.9

The table shows associated comorbidities & other parameters for the total of 115 DS patients included in this study.

From the 107 patients, 427 results of thyroid function tests were collected. Around 86 (80.4%) patients had more than one TFT determination, out of which 62 patients were tested every 3 months. These were patients with SCH. For 19 patients, TFT was done every 6 months. For three patients, the TFT was done in <3 months as they were on thyroxine and was done for drug adjustment. For two patients, the TFT was done every year and for 21 (19.6%) patients repeat test was not done.

Nine patients had transient hypothyroidism (2/11 CH and 7/30 SCH). These patients were not on therapy and had normal TSH and T4 on their follow‐up tests. For the two patients with CH, their TSH was slightly raised but their T4 was in high normal range, they were followed with repeat tests, and it took 6½ months on average for the TFT to normalize in the CH group, whereas TFT took 8 months to normalize in the SCH group. The highest recorded diagnostic TSH for all transient cases was 12.7 μIU/ml. The mean T4 was 11.39 μg/dl. All patients with transient thyroid abnormalities were diagnosed before 1 year of age.

Analysis of common comorbid conditions showed that over half (63/115, 54.8%) of the DS patients had congenital cardiac lesion confirmed with echocardiography, 26 (22.6%) had visual impairment (strabismus), and 7 (6.1%) had seizure disorder and other comorbidities shown in Table 4.

## 5 DISCUSSION

DS is associated with several medical conditions and one of which is thyroid abnormalities.^5^ There is a higher risk of thyroid function deterioration over time in patients with DS which seems to be related to higher baseline TSH levels at diagnosis and autoimmunity. However, apart from overt hypothyroidism, SCH in DS appears to be unrelated to autoimmunity.^26^ DS is usually associated with extra‐thyroidal autoimmunity than thyroid autoimmunity.^27^, ^28^, ^29^ Regardless of the underlying causes, untreated hypothyroidism may aggravate growth and development retardation in patients with DS during infancy and childhood.^22^ Thyroid hormones are vital hormones essential for the central nervous system, particularly during infancy.^20^ The physical signs and symptoms of hypothyroidism overlap with DS. Hence, hypothyroidism may not be considered in infants with DS. This observation leads to have a regular screening of DS patients for possible thyroid abnormalities.^22^

There is a wide range of prevalence of thyroid abnormalities in DS patients which can be explained by the variability in the definition of thyroid disorders, the different population size or the age of patients studied and by the different techniques used to measure the TFT in different studies.^11^ In the current study, thyroid abnormalities were detected in 51 patients (47.7 %), which was higher than studies done in other countries like South Africa (34.5%), California (32.5%) and Oregon (24%).^6^, ^22^, ^25^ Though, AAP recommends screening for thyroid abnormalities in DS patients at birth, 6 months, 12 months, and yearly then after,^4^ in the present study, a significant number of thyroid abnormalities were detected between 2 and 6 months of age. This might result in increased prevalence of thyroid abnormalities in the current study compared with the above studies. Other studies also recommend to have additional testing between 2 and 6 months of age.^6^, ^22^ Therefore, we propose additional screening in children with DS for thyroid abnormalities between 2 and 6 months of age in order to detect the abnormalities earlier and to start treatment timely.

In the current study, boys were affected with thyroid abnormalities predominantly than girls. But there was no statistically significant difference in the mean TSH and T4 between the two sexes unlike the general population where thyroid abnormality is common in girls than in boys.^30^ The same finding was also seen in another study done in Oregon.^6^

Advanced maternal age during conception is a risk factor for DS and other aneuploidy as a result of different speculated mechanisms such as recombination errors in the foetal stages of oogenesis, age‐related accumulation of damaged DNA and age‐related hormonal variations which can increase the prevalence of aneuploidy in advanced age.^31^ In this study, we found more than half of the mothers were older than 35 years of age when they got pregnant with these children substantiating association between older age and increased risk for DS.^31^

SCH was the most common thyroid dysfunction seen in our study which is similar to previous studies done in South Africa, Oregon and India.^6^, ^25^, ^32^ Over expression of interferon which is found on chromosome 21 is one of the suggested mechanisms in causing SCH.^18^ Thyroid autoimmunity is also another postulated cause of SCH.^10^ Unfortunately, we were not able to confirm this fact because of lack of autoantibody tests in our setup. There are many arguments regarding treatment of SCH with levothyroxine and it's believed that there are possible beneficial effects on growth and development in children with DS.^33^ In this study except for patients with transient thyroid abnormalities and children under 1 year of age who had mildly elevated TSH, all children with SCH were treated with levothyroxine as it is recommended in another study.^34^

Despite the burden of both infectious and non‐infectious diseases and inconsistent availability of TFT tests in Ethiopia, TFT was done for 93% of the patients, which was higher compared with another study done in South Africa (83.6%).^25^ The eight patients who had no TFT determination were linked to the endocrine follow‐up clinic from regular outpatient clinic for screening and follow‐up but they did not show up.

In the current study, majority of patients were tested for thyroid function after the neonatal period. This might be the result of delayed diagnosis of DS because of lack of antenatal and newborn screening protocol in our setting. This calls for routine prenatal and neonatal screening of DS and thyroid disorders to avoid delay in diagnosis.

In our study, nine patients with hypothyroidism, both congenital and subclinical, which were not on therapy, had normal thyroid function on follow‐up tests. All of them were below the age of one year. A similar finding was observed in a study done in Oregon^6^ which can be explained by a temporary deficiency of thyroid hormone, which later recovers as a result of improved thyroxine production.^35^ Based on this observation, we suggest follow‐up TFT is important before initiating treatment at least for 6–8 months, especially when the TSH is mildly elevated and the T4 level is in a normal range.

The extra genetic material in DS results in malformations and medical conditions.^36^ In our study, significant number of patients had congenital heart disease (CHD) and visual problems, and few of the patients had sleep apnoea, seizure disorders, autistic spectrum disorders and Hirschsprung disease. These signify the importance of establishing and strengthening DS clinic and the need for a thorough evaluation of DS patients by paediatricians and area experts as these abnormalities further compromise their quality of life.^5^

## 6 CONCLUSION AND RECOMMENDATIONS

In conclusion, thyroid abnormalities were seen in a high proportion of DS patients in our study which calls for placement of regular screening of patients with DS for thyroid abnormalities. The significant detection of abnormalities in the patients in age range of 2–6 months demands the need for additional TFT in this age range apart from the standard recommendation. Patients with DS should be screened also for other common co‐morbidities and linked to respective referral clinics timely to decrease impairment of cognition and improve their quality of life.

## CONFLICT OF INTEREST

The authors declare that they have no competing interests.

## AUTHOR CONTRIBUTIONS

**Birtukan Mulu:** Conceptualization (lead); data curation (lead); formal analysis (lead); funding acquisition (lead); investigation (lead); methodology (lead); project administration (lead); resources (lead); software (lead); visualization (lead); writing – original draft (lead). **Bereket Fantahun:** Conceptualization (supporting); data curation (supporting); formal analysis (supporting); funding acquisition (supporting); investigation (supporting); methodology (supporting); project administration (supporting); resources (supporting); software (supporting); supervision (lead); validation (lead); visualization (supporting); writing – original draft (supporting); writing – review and editing (lead).

## ETHICAL APPROVAL

The research got IRB approval from SPHMMC IRB.

## CONSENT TO PUBLISH

Not applicable.

## Data Availability

All relevant data are available upon reasonable request.
